# Paralogous Genes Involved in Embryonic Development: Lessons from the Eye and other Tissues

**DOI:** 10.3390/genes13112082

**Published:** 2022-11-09

**Authors:** Michaela Drobek

**Affiliations:** Laboratory of Transcriptional Regulation, Institute of Molecular Genetics of the Czech Academy of Sciences, Videnska 1083, 142 20 Praha 4, Czech Republic; michaela.drobek@img.cas.cz

**Keywords:** gene dosage, embryonic development, duplication, paralogous genes, eye

## Abstract

During evolution, gene duplications lead to a naturally increased gene dosage. Duplicated genes can be further retained or eliminated over time by purifying selection pressure. The retention probability is increased by functional diversification and by the acquisition of novel functions. Interestingly, functionally diverged paralogous genes can maintain a certain level of functional redundancy and at least a partial ability to replace each other. In such cases, diversification probably occurred at the level of transcriptional regulation. Nevertheless, some duplicated genes can maintain functional redundancy after duplication and the ability to functionally compensate for the loss of each other. Many of them are involved in proper embryonic development. The development of particular tissues/organs and developmental processes can be more or less sensitive to the overall gene dosage. Alterations in the gene dosage or a decrease below a threshold level may have dramatic phenotypic consequences or even lead to embryonic lethality. The number of functional alleles of particular paralogous genes and their mutual cooperation and interactions influence the gene dosage, and therefore, these factors play a crucial role in development. This review will discuss individual interactions between paralogous genes and gene dosage sensitivity during development. The eye was used as a model system, but other tissues are also included.

## 1. Introduction

The number of genes is organism-specific, with the budding yeast *Saccharomyces cerevisiae* having a total of ~6275 genes [1], the fruit fly ~13,600 genes [2], the chicken ~20–23,000 [3], the dog ~19,000 [4], the pig ~22,342 [5], the mouse ~30,000 [6], and the human ~20,000–25,000 genes. The gene dosage represents the number of copies of particular genes present in the genome. The number of gene copies can be naturally increased by duplication. The creation of an extra gene copy leads to the initial amplification of the gene dosage, which is either further maintained or eradiated. Duplication events include small-scale duplications (SSD), such as tandem or segmental gene duplications, and whole-genome duplications (WGD) that duplicate all the genetic information present in the genome [7]. Genes that have evolved through gene duplication within the same genome are classified as paralogs [8]. Examples of entire gene families being duplicated during evolution include the zinc finger transcription factor (ZNF) subfamily KRAB-ZNF on human chromosome 19 [9], ~200 G-protein-coupled receptors (GPCRs) in humans [10], the serine protease inhibitor (serpin) family [11,12], the mouse and human fibroblast growth factor family (FGF) [13], and the olfactory receptor gene (OR) superfamily [14,15]. Interestingly, during early vertebrate evolution, the ancestral vertebrate underwent two rounds (2R) of WGD [16]. Teleost fish underwent an additional, fish-specific third round (3R) of WGD [17,18]. Multiple rounds of gene duplication allowed the creation of innovative functions during evolution [19].

**Figure 1:**
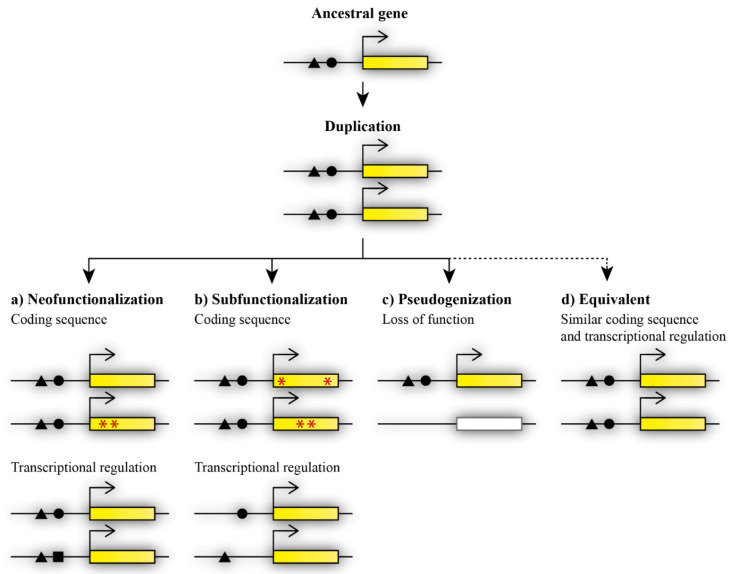
Possible fates of paralogous genes after duplication. Duplicated genes can acquire divergent functions via (**a**) neofunctionalization, in which one duplicate retains the ancestral gene function while the other duplicate acquires a novel function, and the acquisition of the novel function occurs through mutations in the gene coding sequence (indicated by red asterisks) or changes in transcriptional regulation (black square), or (**b**) subfunctionalization, in which mutations change the functions of both duplicates at the level of coding sequence (red asterisks) or transcriptional regulation (missing black triangle or circle), therefore causing the presence of both copies to be essential for the maintenance of the ancestral function. Other possible fates of duplicated genes are (**c**) loss of function during the pseudogenization process, or, less frequently occurring, (**d**) maintained equivalent functions.

Duplicated genes are initially redundant in function. Functional redundancy means that duplicates have the same function, with none or minor phenotypic consequences in the case that one copy is eliminated [20]. The remaining paralogous gene is sufficient to compensate for the loss of the second paralog. Therefore, redundant duplicates provide an ideal source of genetic material that can be used as the origin of new genes [21]. Duplicated genes can follow different fates after the initial duplication (Figure 1). Duplicated genes can be further maintained when it is advantageous for the organism, or eliminated through selective pressure during evolution. Maintained duplicated genes can retain functional redundancy or become functionally divergent. Dosage amplification and back-up compensation contribute to the maintenance of functionally redundant gene copies. Dosage amplification is characterized by an increased number of gene copies, and back-up compensation is characterized by the ability of a paralogous gene to replace the loss of its paralogous partner [21]. However, the acquisition of novel functions increases the probability of retaining duplicated genes [22].

Functional diversification may occur via neofunctionalization or subfunctionalization and can contribute to the maintenance of both paralogous genes [21]. During neofunctionalization, a paralogous gene acquires a new function that was not present in the ancestral genome [21]. The novel function is achieved by the accumulation of mutations that may occur in the coding or regulatory sequence in one of the duplicated genes. The other duplicate thus retains the ancestral function (Figure 1a). Subfunctionalization, as previously described via the duplication–degeneration–complementation (DDC) model, results in the division of the ancestral function between paralogous genes [23]. Each duplicate retains part of the ancestral gene’s function. Mutations may occur in the coding or regulatory sequence at different sites of each duplicate and thus lead to changes in the coding sequence or the spatiotemporal diversification of expression between the duplicated genes [24]. The regulation of gene expression, expression patterns, and changes at the protein level play an important part in the acquired divergent functions after duplication [25,26,27]. In contrast, pseudogenization contributes to the eradication of the redundant copy (Figure 1c). One of the duplicates becomes a null allele, and thus loses its function after duplication [7]. In this case, the extra copy was not maintained, probably due to it not being beneficial for the organism and not improving fitness or specific features of the organism. In general, the loss of an extra copy is more frequent than the maintenance of a functionally redundant copy [20]. Nevertheless, some duplicated genes can maintain initial redundancy during evolution (Figure 1d).

Paralogous genes with a retained function observed within one organism can perform redundant and/or divergent functions. Functional redundancy and divergence between duplicates may vary in different tissues and organs within the same organism [28,29,30,31,32,33]. Moreover, some duplicated genes can perform redundant functions during development, but acquire divergent specific roles in adulthood [34].

The required threshold level may be specific for each tissue and organ, leading to a different sensitivity to the overall amount of the gene product during development. A lower amount of the gene product (below the threshold) can disrupt the proper development and fitness of the developing embryo.

Haploinsufficient genes require the presence of both copies. The loss of one copy reduces the required gene dosage and is linked with many human disorders. For example, the haploinsufficiency of T-box transcription factor 5 (TBX5) leads to congenital heart disease [35]; the haploinsufficiency of proline-rich 12 (PRP12) causes neurodevelopmental and eye diseases [36]; SHANK3 mutations predispose to autism [37]; SETD1A is linked with neurodevelopmental disorders [38]; a SOX5 haploinsufficiency is linked with neurodevelopmental disorders, intellectual disabilities, and language delays [39]; a BAZ1B haploinsufficiency is linked with neurodevelopmental disorders [40]; and the haploinsufficiency of PUM1 causes developmental delays and seizures [41].

In this review, I provide illustrative examples of a functional overlap between paralogous genes involved in embryonic development, with an emphasis on the gene dosage requirements and sensitivity leading to more or less severe alterations in proper embryonic development. The amount of the gene product may be crucial for the development of particular tissues and organs. The sensitivity of particular tissues to the gene dosage and the functional equivalency of paralogous genes were examined by combinations of single and compound losses of paralogous gene functions. The degree of retained functional equivalency was also studied using a series of replacement experiments. Individual sections of this review will discuss the degree of the retained functional equivalency of paralogous genes, the maintained redundancy between paralogous genes, the gene dosage requirements, the sensitivity of particular tissues/organs and processes during embryonic development, and finally, the acquired divergent functions of duplicated genes.

## 2. Retained Functional Equivalency between Paralogous Genes

The functional equivalence between paralogous genes during development was examined using a series of experiments where genes were replaced by their paralogs. The results of the studies revealed that many paralogous genes retained a certain level of functional equivalence and were able to replace each other. Functional equivalence was observed for paralogous genes that function redundantly, but also for paralogous genes with divergent functions. The acquisition of novel expression sites after the initial duplication increased the probability of gene retention and allowed for the acquisition of novel functions, while the functional interchangeability of duplicated genes was mostly maintained [22].

### 2.1. Interchangeable Paralogs with Divergent Functions

Many paralogous genes that had evolved distinct functions maintain a high degree of functional equivalency and are able to replace each other. As an example, the *Myc* family of the cellular oncogenes N-*myc* and c-*myc* are essential for mouse embryonic development, cellular growth, and differentiation. c-*myc* and N-*myc* have broad divergent expression patterns that change during development. Mice with a c-*myc* coding sequence replaced by the N-*myc* coding sequence were viable with normal fertility. Normally, the aberrant expression of c-*myc* leads to embryonic lethality. The N-*myc* expressed from the c-*myc* locus that properly mimicked c-*myc* expression was functionally complementary and regulated similarly to c-*myc*. The differences between the c-*myc* and N-*myc* paralogous genes are probably caused by different transcriptional regulation, leading to distinct specific expression patterns [26]. Divergence driven at the transcriptional level enables paralogs to functionally replace each other after substitution.

As another example, the mouse *Engrailed* genes *Engrailed-1* and *Engrailed-2* have different expression patterns, and their deletion leads to divergent phenotypes. The loss of *Engrailed-1* leads to death after birth with the absence of the major part of the midbrain–hindbrain, while the loss of *Engrailed-2* causes alterations in the cerebellum, without affecting mouse viability. *Engrailed-1* with the coding sequence replaced by the *Engrailed-2* coding sequence is able to compensate for and rescue defects in *Engrailed-1* mutants. The ability to rescue a mutant phenotype by the replacement of paralogous genes suggests that their divergent functions are due to differences in the expression pattern [42]. *Axin* and *Axin2*, which are essential components of Wnt signaling, also exhibit functional interchangeability. The loss of *Axin* leads to early embryonic lethality. The deletion of the paralogous gene *Axin2* leads to a different phenotype; mice lacking *Axin2* are viable with craniofacial defects. However, the expression of *Axin2* under the *Axin* promoter reveals a functional equivalency between *Axin* and *Axin2*, despite their divergent functions in normal conditions. *Axin2* expressed from the *Axin* locus rescued early embryonic lethality in *Axin*-null mice. The absent redundancy between *Axin* and *Axin2* can be a result of their different expression patterns leading to divergent functions [43]. Taken together, different functions of interchangeable paralogous genes are suggested as a consequence of the differing regulation of gene expression and acquired divergent expression patterns.

Interchangeable functions were also observed between coat protein complex II (COPII) components. Coat protein complex II (COPII) vesicles normally mediate transport from the endoplasmic reticulum to the Golgi. The core COPII components *Sec23a* and *Sec23b* acquired distinct functions after duplication. Alterations to their function thus resulted in distinct phenotypes. In humans, the loss of SEC23A leads to an abnormal skeletal phenotype due to the altered secretion of collagen, while the loss of SEC23B affects the development of red blood cells. SEC23B is highly expressed in human bone marrow, but in mice, it is broadly expressed in the pancreas. The *Sec23a* coding sequence placed into the mouse *Sec23b* locus rescued mortality and pancreatic abnormalities in *Sec23b*-null mice. The *Sec23a* coding sequence expressed from the *Sec23b* locus was sufficient to rescue the phenotype in *Sec23b*-null mice, but was not sufficient to rescue the phenotype in *Sec23a*-null mice, suggesting that the exact expression pattern cannot be recapitulated from a different locus [25].

Next, the coat protein complex II (COPII) components *Sec24c* and *Sec24d* play an important role during mouse development. The loss of *Sec24c* causes very early embryonic lethality before the eight-cell stage, while *Sec24d* deficiency results in early embryonic lethality around E7.5. Embryonic lethality caused by the loss of *Sec24c* can be rescued by the *Sec24c* allele with a replacement of 90% of the C-terminal domain of the paralogous Sec24d coding sequence expressed from the *Sec24c* locus. The recombinant allele expressed from the *Sec24c* locus is sufficient to compensate for the loss of *Sec24c*, but insufficient to rescue the phenotype in *Sec24d*-null mice. Probably, the exact expression pattern has not been fully recapitulated when expression occurs in a different locus, or the residual *Sec24c* sequence may impact the ability of functional replacement. In addition, acquired paralog-specific functions could be an explanation for this insufficient compensation. Overall, the experiments showed that *Sec24c* and *Sec24d* maintained functional equivalency and can compensate for each other when they are expressed from the locus of the replaced paralogous gene. The divergence in the *Sec24c*/d protein function is thus probably driven by different tissue- or stage-specific expression [44]. These experiments suggest that the expression pattern can be recapitulated only from the locus of the replaced paralogous gene and that functionally divergent paralogous genes can maintain functional redundancy after duplication even when they normally perform divergent functions.

Additionally, the paired box (Pax) protein coding genes *Pax2* and *Pax5* have divergent expression, with the exception of CNS. Both have overlapping expression in the midbrain–hindbrain boundary. The deletion of *Pax2* or *Pax5* leads to different phenotypes. The loss of *Pax2* causes an absence of a posterior midbrain and cerebellum, while the deletion of *Pax5* results in mild defects in the brain. Nevertheless, both proteins have similar biochemical functions, and *Pax5* expressed from the *Pax2* locus can substitute for *Pax2* functions in the developing ear, eye, and urogenital system. Normally, *Pax5* is not expressed in these tissues. The divergent functions of these paralogous genes are suggested to be a consequence of different timing and levels of their expression. *Pax2* expression starts earlier than expression of *Pax5* during development [45]. The timing of gene expression thus also plays an important role in the exact function of paralogous genes. Biochemical protein properties were probably maintained, and the paralogous gene was thus able to replace and compensate for the phenotypical consequences after the loss of the second paralog.

### 2.2. Common and Unique Functions of Paralogous Genes

Functionally redundant paralogous genes were mostly able to replace each other. The members of the *Cdx* family *Cdx1* and *Cdx2* have a functionally redundant function. *Cdx2* as a substitute for *Cdx1* in *Cdx1*-null mice was able to compensate for *Cdx1* functions. Knock-in mutant mice did not exhibit skeletal defects, the expression of *HOX* genes proceeded normally, and the anterior–posterior vertebral patterning was not altered [46]. *Cdx1* and *Cdx2* were able to replace each other without phenotypic consequences after the replacement, probably due to the maintained functional redundancy.

Nevertheless, the replacement of the orthodenticle homeobox (*Otx)* protein-coding gene *Otx1* by *Otx2* and vice versa exhibited a high degree of similarity, but these genes cannot compensate for each other to the full extent. *Otx2* replaced by *Otx1* is able to function normally in gastrulation and in the rostral neuroectoderm, but not in rostral brain development before the normal onset of *Otx1* expression during development. Replacement led to the loss of anterior structures, including eyes. This indicated that *Otx2* developed novel functions in the anterior neuroectoderm that are not present in *Otx1* [47]. The replacement of *Otx* paralogous genes was also performed between different species. Human *OTX2* expressed from the *Otx1* locus was able to rescue defects in *Otx1*-null mice. The mutant mice did not develop epilepsy, and corticogenesis proceeded normally [48]. Additionally, the human *OTX1* coding sequence expressed under the transcriptional control of *Otx2* had a similar expression pattern as *Otx2*; the mice were phenotypically normal and fertile without defects in the hindbrain and midbrain, and the brain developed normally after the replacement [49]. Paralogous genes with redundant functions were mostly able to replace each other. However, paralogous genes were sometimes not able to replace each other to the full extent, even despite a high degree of functional similarity.

Insufficient compensation was observed in the case that one paralog acquired unique functions. *Pax3* and *Pax7* have partially overlapping expression patterns, which, despite the partial overlap, lead to different phenotypic consequences after their deletion. To investigate their functions, *Pax3* was replaced by *Pax7*. *Pax7* was able to substitute for the functions of Pax3 in the development of the neural tube, neural crest, and somite, but was not able to functionally replace Pax3 in the formation of limb muscles during development. *Pax3* has a unique role in the migration, delamination, and proliferation of muscle precursor cells that cannot be rescued by its paralog *Pax7* [50]. The transforming growth factor β (*Tgfb)* isoforms *Tgfb1* and *Tgfb3* are important for proper embryonic development. The loss of *Tgfb3* results in secondary palate alteration. To test their functional equivalency, *Tgfb1* was inserted instead of the coding sequence of exon 1 in the *Tgfb3* gene. The replacement of Tgf-β3 by Tgf-β1 only partially saved the epithelial fusion defect in *Tgfb3*-null mice. The Tgf-β3 isoform has a specific role in palatal epithelium formation that cannot be compensated for by Tgf-β1 [51].

A similar example comes from Delta-like canonical Notch ligands (Dll); Dll1 and Dll4 have partially redundant functions in myogenesis, fully redundant functions in the maintenance of retinal progenitors, and divergent functions in somitogenesis. Dll4 expressed from the *Dll1* locus in *Dll1*-null mice is able to replace Dll1 in the retina, but is not able to mediate the proper segmentation of the embryo. Only Dll1 is sufficient for proper embryo segmentation; therefore, Dll4 cannot replace the Dll1 functions during embryonic segmentation. The different functions were probably caused by different *cis*-inhibitory properties [31]. Knock-in experiments revealed a partial interchangeability between the members of the *Pax* family genes *Pax6*, *Pax6(5a)*, and *Pax2. Pax6* can be replaced by *Pax6(5a)* or *Pax2* during brain development. The genes have a similar binding specificity and a similar paired domain. However, *Pax6* has acquired a unique role in eye development and cannot be replaced by *Pax6(5a)* or *Pax2*. The substitution of *Pax6* by *Pax6(5a)* or *Pax2* leads to the failure of lens and retinal development [52]. The paralogous genes involved in eye development and their mutant phenotypes are summarized in Table 1.

Paralogous genes were not sufficient to compensate for isoform-specific functions performed by the second paralog. Paralogs retained only partial functional equivalency. The loss of a paralogous gene and its replacement by a second paralog thus led to mild or severe phenotypic consequences.

## 3. Redundant Functions of Paralogous Genes

**Figure 2:**
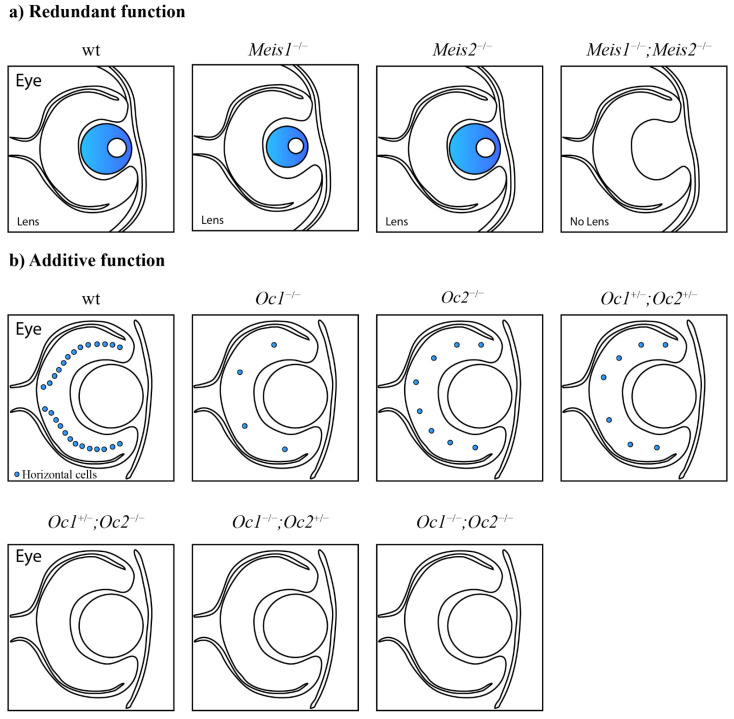

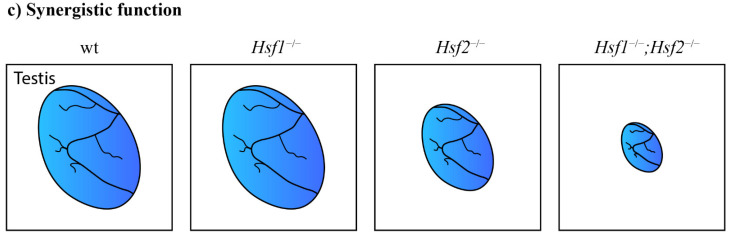
Functional cooperation between paralogous genes. (**a**) Paralogous genes can maintain functional redundancy and the ability to compensate for the loss of each other without phenotypic consequences. *Meis1* can compensate for the lack of *Meis2* and vice versa. Only the combined deletion of paralogous genes results in severe phenotypic alterations. The combined loss of *Meis1* and *Meis2* leads to arrested lens development [60]. (**b**) Paralogs can act additively according to the quantitative model of function. The decreased number of functional alleles causes gradual worsening of the mutant phenotype. Lack of two *Onecut1/Onecut2* alleles in the mouse retina causes a dramatically decreased number of horizontal cells in any combination. One remaining *Onecut1* or *Onecut2* allele, similarly to the combined loss of *Onecut1* together with *Onecut2*, results in complete loss of horizontal cells [62]. (**c**) A synergistic interaction results in a mutant phenotype that exceeds expectations from observations of single knockouts. *Hfs1*-null mice have normal testes, in contrast to *Hsf2*-null mice with decreased testis size. Surprisingly, additional deletion of *Hsf1* in *Hsf2*-null mice causes an even more decreased testis size [76].

Paralogous genes are involved in many essential developmental processes and play a crucial role in the proper development of embryonic tissues and organs. Functionally redundant paralogous genes have maintained a certain level of functional redundancy, and can thus at least partially compensate for the loss of each other. The possible types of cooperation between paralogous genes are shown in Figure 2. Fully redundant paralogs are characterized by their ability to compensate for the loss of the second paralog to the full extent. As examples, studies will be mentioned in the following section.

Dll1 and Dll4 act redundantly in the maintenance of retinal progenitor cells and can functionally replace each other. A fully redundant function was also observed in the maintenance of crypt progenitor cells in the adult small intestine [31]. *Hoxa9* and *Hoxd9* perform fully redundant functions in the formation of the humeral head. *Hoxa9/Hoxd9* compound mutant mice displayed alterations in the shape of the humeral head that were not observed in single *Hoxa9* and *Hoxd9* mutants. *Hoxa9* fully compensated for the loss of *Hoxd9* in the humeral head and vice versa [68]. The Lef/Tcf transcription factors Lef1, Tcf7, Tcf7l1, and Tcf7l2 are important during lung development. A complete redundancy in epithelial progenitor cells during lung development was observed in cases when two or more out of the four paralogous genes were present [69]. The histone deacetylases Hdac1 and Hdac2 have fully redundant functions in maintaining proper chromatin structures that are important during developmental processes [70]. The Nk6 homeobox (*Nkx6)* genes *Nkx6.1* and *Nkx6.2* have fully redundant roles in α-cells during pancreatic development [29,30].

The Sall-like (*Sall*) protein-coding genes *Sall1* and *Sall4* play redundant roles during neural tube development. *Sall1/Sall2* double knockout mice have no obvious alterations in their neural tube development, and *Sall4* was able to compensate for the loss of its paralogous genes in the neural tube [71]. The nuclear Dbf2-related (*Ndr*) family of serine/threonine protein kinases genes *Ndr1* and *Ndr2* function redundantly in cardiac looping and during somite genesis, and can compensate for the loss of each other. Even a single allele of *Ndr1* or *Ndr2* is sufficient for proper heart development and somitogenesis [72]. This is consistent with the fully redundant function between paralogous genes and their ability to compensate for the loss of each other.

Membrane-associated phosphatidylinositol transfer proteins (*Pitpnm*) have also maintained a redundant function. *Pitpnm1* is expressed in the inner ear, and its deletion does not cause hearing defects. Probably, the loss of *Pitpnm1* is compensated for by the paralogous genes *Pitpnm2* and *Pitpnm3* [73]. The transforming growth factor β (Tgf-β) proteins Tgf-β1 and Tgf-β2 act redundantly in secondary palate development in a strain-dependent manner. *Tgfb1* and *Tgfb2* single knockouts have no palate defects in the C57BL/6J background, suggesting that both paralogous genes function redundantly in the C57BL/6J strain. Defects in secondary palate formation were observed in the mixed 129 and Black Swiss strain, suggesting a strain-dependent phenotype in secondary palate development [74]. The maternally imprinted minor splicing factors Zrsr1 (also known as U2af1-rs1) and Zrsr2 (also known as U2af1-rs2) are encoded by the *Zrsr1* and *Zrsr2* paralogous genes. Both genes are essential for zygotic genome activation and both act in a redundant manner. At least one maternal *Zrsr2* or one paternal *Zrsr1* allele is necessary and sufficient for early development [75].

In addition, the homeobox genes *Meis1* and *Meis2* exhibited redundant roles in the lens placode during lens formation [60] (Figure 2a) and in the pool of retinal progenitor cells [61]. The acetyltransferases CBP and p300 displayed redundant roles in lens induction, and even a single functional allele of CBP or p300 was sufficient for lens formation [53]. The fibroblast growth factor receptor genes *Fgfr1*, *Fgfr2*, *Fgfr3*, and *Fgfr4* play a redundant role in lens fiber differentiation [55]. Six3 and Six6 redundantly regulate the maintenance of multipotent neuroretinal progenitors [67].

Fully redundant paralogous genes can compensate for the loss of each other without phenotypic consequences during embryonic development. The deletion of one paralog thus does not lead to phenotypic alterations due to the presence of and functional compensation by the other paralog (Figure 2a).

Nevertheless, paralogous genes can acquire specific functions during evolution and maintain only a partial functional redundancy with their paralog. During embryonic development, partial functional redundancy was observed among many paralogous genes. Some examples include the following: Dll1 and Dll4 have a partially redundant function during embryonic myogenesis [31]. The dishevelled proteins (Dvl) Dvl1, Dvl2, and Dvl3 function partially redundantly during heart development [28]. The Sost domain-containing (*Sost*) paralogous genes *Sost* and *Sostdc1* play partially redundant and complementary roles during limb development, despite their different non-overlapping expression patterns [77]. Odd-skipped related transcription factor 1 (*Osr1*) acts redundantly with *Osr2* in the formation of synovial joints. The deletion of *Osr2* results in only subtle defects in synovial joint development [78]. The immunoglobulin-like cell adhesion proteins nectin1 and nectin3 showed partially redundant functions during tooth development [79].

As another example, the Onecut transcription factors Onecut1 and Onecut2 have a partially redundant function in pancreas organogenesis and in the subsequent steps of pancreatic endocrine differentiation [32] Another function of Onecut transcription factors is discussed in Section 4.1 and Section 5. The transmembrane proteins TMEM120A and TMEM120B function at least partially redundantly during adipocyte differentiation [80]. *Hoxa5* and *Hoxb5* showed partial redundancy during lung morphogenesis [81]. *Hox6* genes have a high degree of functional redundancy in pancreatic organogenesis [82], and the RNA polymerase III subunits Polr3g and Polr3gl are functionally redundant during embryonic development and can partially compensate for each other [83]. Partially redundant paralogous genes perform both common and divergent functions. Therefore, partially redundant paralogs can only partly rescue a phenotype after the loss of the second paralogous gene. Specific functions cannot be compensated for by the other paralogous gene. In general, acquired novel functions are suggested to increase the probability of gene retention, and thus the probability of escape from purifying selection pressure.

## 4. Gene Dosage Sensitivity and Allelic Interactions

Embryonic development can be dramatically influenced by paralogous genes that take part in many developmental processes, and their loss may have severe consequences. The alteration of individual alleles or even the combined loss of paralogous genes changes the level of gene products, which can be critical for the development and maintenance of particular tissues and organs. The required threshold level may differ for individual tissues and organs, leading to different gene dosage sensitivities. The gene dosage required for normal function is thus dependent on the number of functional alleles of paralogous genes. Individual alleles of paralogous genes exhibit different levels of functional redundancy and cooperation, depending on a qualitative or quantitative model. In the quantitative model, they may act additively or even exhibit a synergistic interaction, where the phenotype of the compound mutant is worse than the phenotype that can be expected from individual single knockouts.

### 4.1. Additive Functions

A functional redundancy was observed among *Dlx* gene pairs, *Dlx1*–*Dlx2* and *Dlx5*–*Dlx6*. The *Dlx5* and *Dlx6* paralogous genes exhibited a quantitative model of function in craniofacial morphogenesis. The alleles showed a high degree of functional equivalency and functioned additively. The deletion of *Dlx5* or *Dlx6* had the same phenotypic consequences; both resulted in an alteration to the mandibular arch. Double *Dlx5/Dlx6* heterozygotes also exhibited alterations to the mandibular region. The loss of two *Dlx5/Dlx6* alleles in any combination had similar phenotypic consequences. The deletion of three *Dlx5/Dlx6* alleles led to a markedly reduced or lost proximal dentary structure, whereas the loss of all four alleles caused a transformation of the lower jaw structures to a maxillary identity. The normal pharyngeal patterning without alterations requires a threshold activity of *Dlx5* and *Dlx6*; at least three functional alleles of *Dlx5/Dlx6* must be present. *Dlx5* and *Dlx6* contribute proportionally and additively to craniofacial morphogenesis [84].

Gene dosage sensitivity was also observed in the developing mouse retina. The functionally redundant Onecut transcription factors Onecut1 and Onecut2 function additively in the context of the horizontal cell population (Figure 2b). A single deletion of *Onecut1* or *Onecut2* results in a markedly reduced number of horizontal cells in the mouse retina. A compound *Onecut1/Onecut2* deletion leads to the complete loss of horizontal cells. At least three functional alleles are required for the normal development and maintenance of horizontal cells. A loss of two out of the four functional alleles in any combination leads to a dramatic decrease in horizontal cells. A lack of three or all four alleles results in the complete loss of horizontal cells. Therefore, only one functional *Onecut1* or *Onecut2* allele is not able to rescue the phenotype, and the horizontal cells will be depleted [62].

Next, a functional redundancy in a dosage-sensitive manner was revealed between *Hoxa10*, *Hoxc10*, and *Hoxd10* during kidney development. The combined deletion of any three alleles did not alter normal kidney development. Defects in the kidney development were observed after the deletion of four alleles. The deletion of five alleles led to stronger defects. A lack of six alleles demonstrated the most severe alterations during kidney development; triple knockout mice died after birth. The indistinguishable mutant phenotypes indicate an additive function between the *Hox10* alleles. Kidney development is thus sensitive to the overall *Hox10* gene dosage, requiring the presence of at least three functional alleles for normal kidney development [85]. In general, additively functioning alleles of paralogous genes show a high degree of functional equivalency and exhibit a quantitative model of function (Figure 2b). Particular tissues are sensitive to gene dosage, and thus require a certain number of functional alleles. A gradual decline in functional alleles leads to a gradually worsened phenotype. A drop below the threshold level, which can be different for particular tissues and paralogous genes, has dramatic phenotypic consequences.

Moreover, additive functions between paralogous genes were also observed in Lef/Tcf transcription factors during lung development. Lef/Tcf factors function in the specification of epithelial progenitors. Interestingly, in the presence of at least two out of the four Lef/Tcf transcription factors, Lef/Tcf factors functioned redundantly. Two remaining paralogous transcription factors were sufficient to fully compensate for the loss of their paralogs. Triple Lef/Tcf knockouts displayed varying phenotypes, and quadruple knockouts resulted in underdeveloped lungs and minimal activity of epithelial progenitors. Lef/Tcf factors acted redundantly, but after the initial loss of two, the paralogous Lef/Tcf factors acted additively in the specification and maintenance of epithelial progenitor cells. The redundant and additive functions were dependent on the overall gene dosage. It was also shown that the individual Lef/Tcf transcription factors were not equal. A single remaining Lef1 or Tcfl2 provided partial function in epithelial progenitors, while a single functional Tcf7 or Tcf7l1 provided full function [69]. This research led to the interesting discovery that additive functions can also be observed between non-equivalent paralogous genes.

### 4.2. Synergistic Interaction between Paralogous Genes

Heat-shock factors (Hsf) displayed synergic functions. *Hsf1*-null mice had normal spermatogenesis, while *Hsf2*-null mice had smaller testes and a mild impairment of fertility. Defects in compound mutants were unexpectedly more severe than in single *Hsf2*-null mice (Figure 2c). The deletion of both *Hsf1/Hsf2* factors caused defects in spermatogenesis that led to male sterility. *Hsf* activity is thus essential for normal spermatogenesis and fertility [76]. Additionally, an examination of *Hsf4*-null mice showed that the testes were not affected by an *Hsf4* deletion [58]. The loss of *Hsf4* in the testis was probably compensated for by other paralogous genes. Interestingly, the paralogous *Hsf* genes acquired a stage-specific expression in the lens. *Hsf1* and *Hsf2* are expressed in the fetal lens. The deletion of *Hsf1* or *Hsf2* had no phenotypic consequences in the lens, probably due to compensation by a paralogous gene. In contrast to *Hsf1* and *Hsf2*, *Hsf4* is predominantly expressed postnatally and regulates the expression of αβ-crystallins [59]. The deletion of *Hsf4* led to cataracts and abnormal lens fibers [58]. The *Hsf* family member *Hsf3* acquired unique functions in mice to protect the cell from stress by activating nonclassical heat-shock genes. In humans, *Hfs3* has become a pseudogene [86].

Synergistic interactions were also observed during axial skeleton development. A lack of *Pax1* or *Pax9* resulted in divergent phenotypes. The deletion of *Pax1* resulted in morphological alterations to the axial skeleton, while the deletion of *Pax9* did not cause axial skeleton defects. Nevertheless, the additional deletion of *Pax9* in *Pax1*-null mice resulted in a more severe phenotype. Compound *Pax1/Pax9* mice lost their medial derivates of sclerotomes, vertebral bodies, invertebral discs, and proximal parts of the ribs. Cooperation between *Pax1* and *Pax9* is dosage-dependent; the deletion of three functional alleles causes intermediate phenotypes. The worsened phenotype in *Pax1*-null mice after the additional deletion of *Pax9* revealed a synergistic interaction between *Pax1* and *Pax9* [87]. A synergistic interaction was also observed in the *Hox3* gene cluster. Single *Hoxa3* and *Hoxd3* mutants had no defects in the formation of their cervical vertebrae. The deletion of *Hoxb3* caused slight defects in the formation of cervical vertebrae with low penetrance. Surprisingly, the combined loss of *Hoxa3/Hoxd3* or *Hoxb3/Hoxd3* caused a loss of the entire atlas, suggesting a synergic function of *Hox3* genes. *Hox3* alleles exhibit dosage-dependent interactions and interact in a quantitative manner. The removal of any of the three *Hox3* alleles led to the partial loss of the atlas, whereas the loss of four alleles caused a more severe phenotype, the loss of the entire atlas. Compound *Hoxa3/Hoxd3* and *Hoxb3/Hoxd3* mutants exhibited the same cervical vertebrae alteration as *Hoxa3*^+/−^/*Hoxb3*^+/−^/*Hoxd3*^−/−^ triple mutants. Indistinguishable defects observed after the loss of different allele combinations indicated an equivalent function of the *Hox3* paralogous genes in the cervical vertebrae [88]. A synergistic interaction is characterized by an unexpected mutant phenotype that cannot be expected from observations of single knockouts (Figure 2c). The additional deletion of a paralogous gene in a single knockout leads to more severe phenotypic consequences, despite the fact that the single knockout of the additionally deleted paralogous gene does not have phenotypic consequences.

As another example of synergistic interaction, I can mention *Dvl* genes. The deletion of individual *Dvl1*, *Dvl2*, and *Dvl3* genes results in divergent alterations. A lack of *Dvl1* leads to abnormalities in social interactions and altered sensorimotor gating. A lack of *Dvl2* causes cardiac outflow tract abnormalities and rib and vertebral malformations with incomplete penetrance. The loss of *Dvl3* leads to defects in the inner ear, neural tube defects, and cardiac outflow tract abnormalities causing perinatal lethality. Nevertheless, *Dvl* genes have overlapping expression patterns and exhibit functional redundancy even despite their distinct mutant phenotypes. The additional loss of *Dvl* paralogous genes causes more severe phenotypes, suggesting gene-dosage sensitive redundant roles of *Dvl* genes. A single *Dvl* knockout did not display neural closure defects. The combined loss of three or all four alleles of *Dvl1/Dvl2* or *Dlv2/Dvl3* resulted in neurulation defects. Neurulation in the compound *Dvl1/Dvl3* mutant proceeded normally. Probably, the alleles are not functionally equivalent. Gene dosage sensitivity was also observed in the organ of Corti. Single *Dvl2* and *Dvl3* heterozygotes had normal development of the organ of Corti, whereas double heterozygotes displayed alterations to the development of the organ of Corti. The additional deletion of the *Dvl2* allele in *Dvl3*-null mice worsened the phenotype. A dosage-sensitive function was also observed in cardiac development. Double *Dvl2/Dvl3* heterozygotes exhibited cardiac defects, in contrast to single *Dvl2* and *Dvl3* heterozygotes with normal cardiac development. A more severe phenotype was observed after the deletion of three *Dvl2/Dvl3* alleles. A non-equivalent function was observed in rescue experiments. An extra copy of *Dvl1* was not able to restore cardiac defects in *Dvl2*-null mice, whereas an additional copy of *Dvl1* and *Dvl2* was able to compensate for the loss of *Dvl3* during heart development [28]. Synergistic interactions can thus be observed between equivalent, but also between non-equivalent, paralogous genes.

## 5. Functionally Divergent Paralogs

Naturally, there are a number of instances in which the paralogous genes have acquired distinct functions. For example, the loss of the *homeobox (HOX)* gene *Hoxc-4* does not affect the cervical vertebrae, in contrast to its paralogous genes *Hoxa-4*, *Hoxb-4*, and *Hoxd-4*, but leads to abnormalities in the thoracic vertebrae [89]. *Hoxa10* and *Hoxd10* act independently in the regulation of lumbar and sacral axial patterning. Mutations in *Hoxa10* are the cause of lumbar vertebral transformations, while *Hoxd10* mutations affect the sacral vertebrae [90]. H3K4 methyltransferase MLL3 (also known as KMT2C, lysine methyltransferase 2C) diverged from its paralog MLL4 (also known as KMT2D, lysine methyltransferase 2D). MLL3 is essential for lung maturation, while MLL4 is required for the initiation of gastrulation via the regulation of anterior visceral endoderm migration [91].

As another example, the Onecut transcription factor Onecut1 functionally diverged from its paralogous genes *Onecut2* and *Onecut3.* Only *Onecut1* is required for the specification and morphogenesis of the pancreas and endocrine differentiation. Moreover, *Onecut2* and *Onecut3* are not required for gut development and enteroendocrine differentiation, in contrast to *Onecut1* [32]. Serine protease (Prss) 55, in contrast to its paralog *Prss51*, is essential for male fertility, sperm migration, and binding to the zona pellucida [92]. *Sost* and *Sostdc1* diverge in the majority of organ systems, including the nervous, cardiovascular, musculoskeletal, respiratory, reproductive, and digestive systems [77]. Hdac1 and Hdac2 have functionally diverged during evolution. Hdac2 gained a new function in the regulation of neural precursor cells in brain development during evolution, which is not present in its paralog Hdac1. A single remaining functional allele of *Hdac1* is not sufficient for proper brain development, in contrast to *Hdac2* [70]. The family with sequence similarity 170 member A (*Fam170a*) is important for male fertility, while the paralogous gene *Fam170b* is not essential for male fertility [93]. Paralogous genes that did not maintain functional redundancy underwent a subfunctionalization or neofunctionalization process after the initial duplication. Functional diversification is advantageous due to the increased probability of their maintenance.

Distinct functions emerged in the H3K4 methyltransferase paralogs of the SET-containing domain (Setd), Setd1a and Setd1b. Setd1a is important for gastrulation, whereas Setd1b is dispensable. Setd1b is required later in embryonic development during organogenesis [94]. The winged-helix/forkhead transcription factors (Fox) Foxa1 and Foxa2 play a redundant role during development, but have evolved distinct roles in the adult liver [34]. The TATA-binding-associated proteins (TAFs) TAF4b and TAF4 evolved different roles in regulating the maintenance and proliferation of mouse embryonic stem cells. TAF4b is highly expressed in embryonic stem cells (ESCs), supporting proliferation and progression through the cell cycle. A high proliferation rate is a specific sign of embryonic stem cells. TAF4 has the opposite effect to TAF4b and reduces the growth of ESCs. The expression of TAF4 is essential in later developmental stages [95]. The anti-silencing function 1 (Asf1) histone chaperone genes *Asf1a* and *Asf1b* acquired divergent functions during evolution. The loss of *Asf1a* leads to embryonic lethality, while *Asf1b*-null mice are viable, but exhibit decreased reproductive capacity compared to the wild type [96]. Further studies have shown that *Asf1a* is involved in histone H3.3 deposition in the paternal pronucleus after fertilization and regulates levels of *Oct4* expression and H3K56ac. *Asf1b* is required for the regulation of cell proliferation in early embryos [97]. Divergent paralogous genes can be involved in distinct temporal stages during development, and thus exhibit temporal functional diversity.

Divergences in expression patterns and the performance of distinct functions have been observed between some paralogous genes. For example, *HOXA* cluster genes have expression patterns differing from *HOXC* and *HOXD* in the human endometrium. HOXA10 and HOXA11 regulate the differentiation of the endometrium, whereas HOXC10, HOXC11, HOXD10, and HOXD11 play a role in the regulation of endometrial proliferation [98]. The H6 homeobox genes (*Hmx*) *Hmx1* (also known as *Nkx5-3*), *Hmx2* (*Nkx5-2*), and *Hmx3* (*Nkx5*-1) are expressed in the developing nervous system. *Hmx1* acquired a developmental role differing from its paralogs *Hmx2* and *Hmx3* and is expressed in the eye lens and retina during development. *Hmx2* and *Hmx3* share a similar expression pattern in the developing central nervous system and inner eye [56]. Divergent functions of duplicated genes ensure a higher chance of their maintenance during evolution and escape from the purifying selection pressure leading to elimination of the extra gene copy over time.

Another interesting group of genes to mention are the opsins, which represent G-protein-coupled receptors (GPCRs). There are three opsins present in human cones: opsin1 long-wave-sensitive (OPN1LW, L-cones) and opsin1 medium-wave-sensitive (OPN1MW, M-cones), both localized on chromosome Xq28, and opsin 1 short-wave-sensitive (OPN1SW, S-cones), localized on chromosome 7. Cones are responsible for color vision and the recognition of red (L-cones), green (M-cones), and blue (S-cones) color. Alterations in both OPN1LW and OPN1MW lead to a recessive X-linked disorder called blue cone monochromacy (BMC). The symptoms include dramatically reduced central vision, impaired color vision, photophobia, and congenital nystagmus [63,64]. X-linked disorders are more pronounced in males due to the presence of a single X-chromosome inherited from the mother. The misalignment of opsins during the recombination of two X-chromosomes in females can lead to deuteranopia (green blindness), where the gene coding for the M pigment is replaced by the L gene, or protanopia (red blindness), where the L gene is replaced by the M gene [65]. Individuals with unaltered color vision have a single red pigment gene and one or more copies of the green pigment gene. However, only the most proximal green pigment gene is expressed [99,100]. Compared to humans, the mouse retina consists of only two types of cones, M-cones and S-cones [101].

Interestingly, paralogous genes may have redundant, but also divergent, functions within the same organism. For example, during pancreatic development, *Nkx6.1* and *Nkx6.2* have redundant roles in α-cell development, but distinct roles in β-cell development due to a divergent expression pattern [29,30]. *Dvl1* and *Dvl2* have redundant roles with *Dvl3* in cardiac development, whereas their roles diverge in skeletal development. *Dvl1* and *Dvl2* act redundantly, and their deletion results in skeletal defects, while the loss of *Dvl3* does not cause skeletal defects [28]. Dll1 and Dll4 have partially redundant functions in myogenesis and fully redundant functions in the maintenance of retinal progenitors, but have acquired divergent roles in somite segmentation [31]. The Onecut1 and Onecut2 transcription factors have redundant roles in the mouse retina, but divergent functions in endocrine differentiation and gut development [32,33]. Sometimes, redundancy/divergence between paralogous genes can switch during development. This phenomenon was observed in the case of *Foxa1* and *Foxa2*, which play a redundant role during liver development, but acquire divergent functions in the adult liver [34].

## 6. Conclusions

Embryonic development can be dramatically influenced by paralogous genes and their functional cooperation. Specific developmental processes require the presence of a certain paralogous gene due to its acquired divergent role that cannot be functionally compensated for by a functionally divergent paralogous gene. Interestingly, replacement experiments have revealed the maintained ability of some functionally distinct paralogous genes to replace each other when expressed from the locus of the replaced paralog. This indicates that changes between interchangeable paralogous genes occurred at the level of transcriptional regulation. The biochemical protein properties were probably maintained, and thus sufficient replacement is enabled. Functional interchangeability was observed, for example, between the N-*myc* and c-*myc* [26], *Engrailed1* and *Engrailed2* [42], *Axin* and *Axin2* [43], *Sec23a* and *Sec23b* [25], *Sec24c* and *Sec24d* [44], *Pax2* and *Pax5* [45], and *Cdx1* and *Cdx2* [46] paralogous genes. Nevertheless, some acquired unique paralog-specific functions remain irreplaceable. Irreplaceable specific functions were observed, for example, in *Otx2*, which acquired a specific irreplaceable role in rostral brain development [47]; *Pax3*, which has a unique function in muscle precursor cells [50]; *Tgf-β3*, which acquired a specific role in the formation of the palatal epithelium [51]; *Dll1*, which is required for proper embryo segmentation [31]; and *Pax6*, which acquired a unique role in eye development [52]. The functional diversification of paralogous genes increases the chance of their maintenance. Otherwise, the extra copy is eliminated by purifying selection pressure. A loss of function occurs during the pseudogenization process and is more common than conserved redundancy.

Despite this fact, some paralogous genes can maintain functional redundancy after the initial duplication. Functional cooperation and interactions between maintained paralogous genes influence the gene dosage that is essential for the proper development of particular tissues/organs and in developmental processes. Paralogs with a maintained functional redundancy can compensate for the absence of each other. Phenotypic consequences can be observed in cases where both paralogous genes are missing. Fully redundant functions were observed between the *Dll1* and *Dll4* [31]; *Hoxa9* and *Hoxd9* [68]; Lef/Tcf transcription factors [69]; *Hdac1* and *Hdac2* [70]; *Nkx6.1* and *Nkx6.2* [29,30]; *Sall1* and *Sall4* [71]; *Ndr1* and *Ndr2* [72]; *Pitpnm1*, *Pitpnm2*, and *Pitpnm3* [73]; *Tgfb1* and *Tgfb2* [74]; *Zrsr1* and *Zrsr2* [75]; *Meis1* and *Meis2* [60,61]; *CBP* and *p300* [53]; *Fgfr1*, *Fgfr2*, *Fgfr3*, and *Fgfr4* [55]; and *Six3* and *Six6* paralogous genes.

Additive functions lead to a gradually worsened phenotype with a gradually decreased number of functional alleles of paralogous genes. A gradually decreased number of functional *Dlx5/6* alleles leads to a gradually worsened craniofacial morphogenesis [84]; gradually reduced *Onecut1/Onecut2* alleles cause a dramatically reduced number of horizontal cells in the mouse retina [62]; a gradually decreased number of functional *Hoxa10*, *Hoxc10*, and *Hoxd10* alleles results in gradually worsened kidney development [85]; and a decreased number of *Lef*/*Tcf* alleles gradually alters the specification and maintenance of epithelial progenitor cells [69]. The threshold level of functional alleles is specific for particular tissues and organs, and may differ for individual paralogous genes. A decline below the threshold level results in dramatic phenotypic consequences. The loss of more than one functional *Dlx5/Dlx6* allele leads to alterations in pharyngeal patterning [84], a single *Onecut1*/*Onecut2* allele in any combination is not sufficient to prevent the complete loss of horizontal cells [62], less than three functional *Hoxa10/Hoxc10/Hoxd10* alleles leads to defects during kidney development [85], and the presence of less than two out of the four Lef/Tcf transcription factors results in defects during lung development [69]. Additive functions were mostly observed for functionally equivalent paralogous genes, but non-equivalent paralogous genes may also exhibit additive functions. For example, Lef/Tcf factors function additively in the maintenance and specification of epithelial progenitor cells, but are not equal. A single remaining Lef1 or Tcfl2 factor provides partial function in epithelial progenitors, while a single functional Tcf7 or Tcf7l1 factor provides full function [69].

Next, synergistic interactions lead to unexpected phenotypes. A single knockout itself may have no phenotypic consequences, but the additional deletion in a single knockout of a paralogous gene can unexpectedly cause more severe alterations. The severity of phenotypic consequences thus cannot be predicted from the phenotypes of single knockouts. Unexpected synergistic interactions were observed between *Hsf1* and *Hsf2* [76], *Pax9* and *Pax1* [87], *Hoxa3/Hoxd3* and *Hoxb3/Hoxd3* [88], and *Dvl* paralogous genes [28]. The evolved cooperation and interactions between paralogous genes and their alleles thus play an essential role during proper embryonic development.

Although, in general terms, some predictions of paralog function can be inferred from the known functions of the original gene and the expression profile of the paralog in question, it is still difficult to predict the function of diversified paralogs with certainty. Even with the gradual accumulation of our knowledge, it is unreliable to use, for example, ortholog conjecture in the prediction of gene function [102], not to mention the dependency of paralogs or their compensatory capability for each other [103,104]. Thus, the most reliable approach still remains tedious experimental work on model organisms.

## Figures and Tables

**Table 1:** Overview of paralogous genes involved in eye development.

Gene Name	Chromosomal Location		Number of Exons		Length of Protein (AA)		Protein Identity (%)	Phenotypes
	Mouse	Human	Mouse	Human	Mouse	Human		
*CBP*	16 A1	16p13.3	31	33	2441	2442	95.57	Altered lens induction [53]
*P300*	15 E1	22q13.2	31	31	2412	2414	94.22	
*Dll1*	17 A2	6q27	11	11	722	723	88.77	Affected neurogenesis and maintenance of RPCs [31,54]
*Dll4*	2 E5	15q15.1	11	11	686	685	86.42	
*Fgfr1*	8 A2	8p11.23	28	24	822	822	98.42	Altered lens fiber differentiation [55]
*Fgfr2*	7 F3	10q26.13	19	26	821	821	96.95	
*Fgfr3*	5 B2	4p16.3	20	19	801	806	93.12	
*Fgfr4*	13 B1	5q35.2	19	18	799	802	89.86	
*Hmx1*	5 B3	4p16.1	3	3	332	348	89.46	Ophthalmic anomalies [56,57]
*Hmx2*	7 F3	10q26.13	3	2	273	273	93.77	No eye-specific phenotype, hearing loss [56]
*Hmx3*	7 F3	10q26.13	5	2	356	357	96.07	
*Hsf1*	15 D3	8q24.3	14	15	525	529	89.71	*Hsf4*^−/−^ cataract and abnormal lens fibers [58,59]
*Hsf2*	10 B4	6q22.31	14	13	535	536	94.76	
*Hsf4*	8 D3	16q22.1	13	15	492	492	86.76	
*Meis1*	11 A3	2p14	14	13	390	390	99.74	Arrested lens formation [60], hypocellular retina [61]
*Meis2*	2 E4-E5	15q14	19	15	477	477	99.58	
*Onecut1*	9 D	15q21.3	2	3	465	465	98.92	Loss of horizontal cells [62]
*Onecut2*	18 E1	18q21.31	3	6	505	504	99.01	
*Opn1lw*	-	X8q28	-	6	-	364	-	Blue cone monochromacy (BMC) [63,64], deuteranopia, protanopia [65].
*Opn1mw*	X A7.3	X8q28	6	6	359	364	87.74	
*Opn1sw*	6 A3.3	7q32.1	5	5	346	348	85.26	
*Pax6*	*2 E3*	11p13	16	25	422	422	100	Failure of lens and retinal development [52], ocular defects, incomplete fissure closure [66]
*Pax2*	19 C3	10q24.31	13	14	414	417	99.03	
*Six3*	17 E4	2p21	3	2	333	332	97.59	Defects in maintenance of multipotent neuroretinal progenitors [67]
*Six6*	12 C3	14q23.1	2	2	246	246	97.97	

Chromosomal locations and numbers of exons were obtained using the NCBI Gene website. Protein length and similarity between orthologous genes were obtained via the Uniprot website.
